# Epidemic Erysipelas—A Report Read before the Medical Society of South-Western New York

**Published:** 1865-05

**Authors:** Thos. D. Strong

**Affiliations:** Westfield, N. Y.


ART. IV.—*Epidemic Erysipelas—A Report read before the Medical Society of South-Western New York, by* THOS. D. STRONG, M. D., *Westfield, N. Y.*

During the last half of February, March, and first week in April, an epidemic occurred in the town of Ripley, in this county, of great interest, both from the severity of the attacks and from the variety and severity of the complications. No age was exempt, though it was generally confined to adults, and was mostly within the area of twenty-four miles. A few cases occurred in the adjoining towns of Westfield and North East.

The disease commenced with a distinct chill; sometimes as severe as that of an intermittent, followed by violent pain in head and general aching of the bones. When reaction occurred (in a few hours), there was heat of skin, flushed face, great thirst, entire loss of appetite, pulse from 110 to 140, soreness of throat, the whole pharynx being actively inflamed, in many cases the tonsils were swollen so as to touch each other, in others swollen scarcely any; in many the swelling externally at angle of jaw was great, apparently involving the parotid and submaxillary glands; some complained of soreness of the muscles of neck laterally and behind.

The early appearance of the throat and the constitutional disturbance indicated diphtheria, and I watched, expecting to see false membrane, but none appeared.

The first case I saw had been sick five days. She then had erysipelas of face, which finally involved the whole head; was 56 years old; had great difficulty of deglutition; tongue dry and black; teeth covered with sordes, delirious; skin hot and dry, and great irritability of stomach, so that it scarcely retained anything for a time. The next nine cases were those who had taken care of her, or the sick ones who had been with her. Afterwards it showed itself in families a mile or two distant, and where no one had seen a case of this sickness. In one of these families there were six cases, and in another four, one of them fatal.

The complications mostly occurred in three to eight days, after a marked mitigation of the throat troubles and constitutional disturbance. One case had acute glossitis, six had acute bronchitis, three had pneumonia, five had acute rheumatism, six had erysipelas, four of the head, one of arm, one of hand. One had inflammation of supra-orbital cells, antrum and ear, and two had peritonitis. These last two had each taken an active cathartic by domestic prescription. One died—she was a healthy woman of forty-five years of age. Saw her first after the peritonitis was declared. The soreness of throat was much better, though the swelling at angle of jaws remained, and she could open the mouth but partially. The peritonitis was declared thirty-six hours before, by chill and vomiting, followed by acute pain, tenderness of abdomen, and intense tympanitis. Her position was upon her back; shoulders raised and knees drawn up. Her treatment was morphine, verging to narcotism, quinine, ess. beef, and warm fomentations. She died in five days from the onset.

Her daughter, about nineteen years old, had been absent, and returned the day of her mother's death. In four days after, she was taken with a severe grade of the throat trouble, and in three days more with erysipelas of the head. Her pulse was 140; delirious; tongue dry and black; teeth covered with sordes; urine passed involuntarily; head immensely swollen. She recovered.

In no case did suppuration occur either in tonsils or the external swelling of neck.

In all the cases both mild and severe, the patients complained of great debility. The constitutional disturbance seemed greatly disproportioned to the local trouble where severe complications did not occur. I was early struck with the evident signs of blood-poisoning, and the strong epidemic (if not contagious) tendency of the disease, and in the first case the typhoid symptoms were so evident that no one could mistake them.

I will say but few words about the treatment; it was nearly the same in all, varying more in *degree* than *kind*. A cardinal point was to sustain; quinine—from 6 to 20 grains daily; whisky—from teaspoonful every two hours to tablespoonful every hour; ess. beef, chicken broth and milk porridge *ad libitum*. In all the cases either chlorate of potash or sesquichloride of iron were used; anodynes were used moderately.

The condition of the patients, the evident blood-poisoning, and the typhoid tendency of the complications, pointed to this course of treatment; and the results would alike justify it, as but *one died*, and that *one* not having treatment until thirty-six hours after peritonitis was fully declared.

This report is based upon notes taken in thirty-six cases in my own practice.

